# Combined Oral Contraceptives and Vascular Thrombosis: A Single-Center Experience

**DOI:** 10.7759/cureus.25865

**Published:** 2022-06-12

**Authors:** Mohammed AlSheef, Yacoub Abuzied, Ghady R Alzahrani, Nihal AlAraj, Nada AlAqeel, Hala Aljishi, Mukhtar J Alomar, Abdul Rehman Z Zaidi, Ohoud M Alarfaj

**Affiliations:** 1 Internal Medicine and Thrombosis, Medical Specialties Department, King Fahad Medical City, Riyadh, SAU; 2 Nursing Department, Rehabilitation Hospital, King Fahad Medical City, Riyadh, SAU; 3 Pharmacy Department, Prince Sultan Military Medical City, Riyadh, SAU; 4 Pharmacy Services Department, Prince Mohammed Bin Abdulaziz Hospital, Riyadh, SAU; 5 Pharmaceutical Care Department, King Abdullah Specialist Children’s Hospital, King Abdulaziz Medical City, Riyadh, SAU; 6 Epidemiology and Public Health, King Fahad Medical City, Riyadh, SAU; 7 Pharmacy Services Administration, King Fahad Medical City, Riyadh, SAU; 8 Internal Medicine, Medical Specialties Department, King Fahad Medical City, Riyadh, SAU

**Keywords:** women, pe, dvt, combined oral contraceptives, thrombosis

## Abstract

Background

Combined oral contraceptives (COCs) are frequently prescribed for contraception, to regulate ovulation and treat endometriosis, and to control menopausal symptoms. A major risk of hormonal contraceptives is vascular thrombosis.

Methods

A retrospective chart review of female patients with deep vein thrombosis (DVT), pulmonary embolism (PE), or other sites of thrombosis or emboli seen in the thrombosis clinic of the department of internal medicine at a tertiary care hospital in Saudi Arabia between March 2010 and February 2015 was performed to identify and characterize which women were taking COCs.

Results

Of 1,008 patients treated for DVT, PE, or other sites of thrombosis or emboli, 100 (9.9%) were taking COCs. Venous (98%) and arterial (2%) thromboses were seen. Overall, 62% of the patients experienced a DVT and 26% pulmonary emboli, and 20% of the patients experienced unusual sites of thrombosis. Furthermore, 53% were obese or morbidly obese. The incidence of venous thrombosis was the highest during the first year of COC use (73%). Of the patients, 8% had thrombophilia.

Conclusion

This study characterizes Saudi women with thrombotic events taking COCs and identifies risk factors, including unusual sites of thrombosis. Most patients experienced the vascular event during the first year of taking COCs. Age of 40-50 years, obesity, and thrombophilia were the commonly observed risk factors.

## Introduction

The most common vascular cause of death after stroke and myocardial infarction (MI) is venous thromboembolism (VTE) [1]. More than 600,000 cases of deep vein thrombosis/pulmonary embolism (DVT/PE) and 50,000 deaths due to PE occur yearly in the United States [2]. The long-term complications of these events include recurrent DVT, recurrent PE, post-thrombotic syndrome, and chronic thromboembolic pulmonary hypertension [3].

Combined oral contraceptives (COCs) are the major treatment used to prevent ovulation, implantation, and pregnancy [4]. They are also used in the treatment of polycystic ovarian syndrome and endometriosis [5]. One of the major adverse effects of COCs is the increased risk of VTE. Overall, the use of COCs is associated with a fourfold to sevenfold increase in the risk of venous thromboembolic events [6-9]. However, the use of COCs can also increase the risk of arterial thrombosis by twofold to fourfold [9,10]. If COCs are combined with a heterozygous factor V Leiden mutation, the risk of venous thrombosis is increased up to 35-fold, while 16-fold if combined with prothrombin G20210A mutation [11,12].

It is important to note that the risk of thrombosis is not only attributed to the use of oral contraceptive pills but also to other means of contraception, such as the use of transdermal patches or vaginal rings [13,14]. There is also an increased risk of VTE in patients using COCs as they age. A study published in 2011 concluded that the adjusted relative risk increased 6.8-fold from the youngest (15-19 years old) to the oldest (40-49 years old) age category of women [15]. Smoking also increases the risk of venous and arterial thrombosis and acts synergistically with oral contraceptive use [16,17].

We conducted this study to describe the clinical and demographic characteristics of patients with COC-associated vascular thrombosis in Saudi Arabia.

## Materials and methods

A five-year retrospective chart review of female patients seen in the thrombosis clinic of the Department of Internal Medicine at King Fahad Medical City (KFMC), Riyadh, Saudi Arabia, from March 2010 to February 2015 was performed. Ethical approval was provided by the KFMC Human Research Ethics Committee (approval #15-205E). Women with DVT, PE, or other sites of thrombosis or emboli who were taking COCs at the time of the event were identified. The diagnosis was confirmed by Doppler ultrasonography or computed tomography.

A case report form was used to collect data from patient charts. Data extracted included demographic information, type of COC, length of time taking COCs, diagnosis, risk factors for VTE, patient adherence to anticoagulant therapy, and history of previous VTE. Different risk factors for VTE were evaluated, including patient age, obesity (body mass index (BMI) > 30 kg/m^2^ calculated as weight (kg)/height (m)^2^), hypertension, diabetes, cancer, personal or family history of VTE (father, mother, sister, or brother), and history of recent surgery or hospitalization. The collected data was checked for completeness and accuracy by a second reviewer.

Thrombophilia testing evaluated activated protein C resistance (APCR), factor V Leiden mutations, lupus anticoagulant (LA), protein C deficiency, protein S deficiency, antithrombin III (ATIII) deficiency, JAK2 mutation, prothrombin gene mutation G20210A, antiglycoprotein antibodies, and anticardiolipin antibodies.

Statistical analysis

Baseline characteristics were presented as the mean±standard deviation (SD) for continuous variables and count (percentage) for categorical variables. Categorical variables were analyzed using chi-square tests. All statistical tests were two-tailed. P<0.05 was considered statistically significant. Data were analyzed using SPSS version 22 (IBM Corp., Armonk, NY, USA).

## Results

A total of 1,008 female patients were treated for thrombosis or emboli over the five-year period. Of these patients, 100 (9.92%) were also taking COCs (Table 1). The mean patient age of women taking COCs was 34±8.1 years. Of the patients, 44 (44%) were greater than 35 years of age. All patients were Saudi, and 98 (98%) were married.

**Table 1:** Patient Characteristics

Patient Characteristics		N (%)
Age (years)	35>	56 (56)
	>35	44 (44)
BMI	Underweight	1 (1)
	Normal	15 (15)
	Overweight	31 (31)
	Obese	45 (45)
	Morbidly obese	8 (8)
Nationality	Saudi	100 (100)
Marital status	Married	98 (98)
	Not married	2 (2)
Use of combined oral contraceptives		100 (100)
Type of COC	Diane (cyproterone acetate 2 mg and ethinyl estradiol 0.035 mg)	6 (6)
	Gynera (gestodene 0.075 mg and ethinyl estradiol 0.03 mg)	18 (18)
	Marvelon (ethinyl estradiol 0.035 mg and desogestrel 0.15 mg)	14 (14)
	Yasmin (drospirenone 3 mg and ethinyl estradiol 0.03 mg)	8 (8)
	Unknown	54 (54)

Information about the time interval between the start of COC use and the development of VTE/PE was available in 53 (53%) patients. Of the charts where the time interval was known, women with VTE/PE used COCs for an average of 13.6±2.7 months. Eighteen (34%) patients had used COCs for less than three months, 13 (24.5%) for 3-6 months, eight (15.1%) for 7-12 months, and 14 (26.4%) for more than 12 months. The type of COC used was known in 46% of patients, mainly third-generation products (Diane (cyproterone acetate 2 mg and ethinyl estradiol 0.035 mg), n=6 (6%), Gynera (gestodene 0.075 mg and ethinyl estradiol 0.03 mg), n=18 (18%), Marvelon (ethinyl estradiol 0.035 mg and desogestrel 0.15 mg), n=14 (14%)) and to lesser extent fourth-generation products (Yasmin (drospirenone 3 mg and ethinyl estradiol 0.03 mg), n=8 (8%)).

Factors that were found to be associated with VTE/PE included obesity or morbid obesity (53%), recent surgery (15%), family history of VTE (9%), thrombophilia (8%), immobilization (8%), history of previous VTE (7%), and diabetes (5%). Evaluation for thrombophilia identified five (5%) patients with protein S deficiency, one (1%) with a factor V Leiden mutation, one (1%) with a combined protein C and S deficiency, and one (1%) with a combined factor V Leiden mutation and protein S deficiency (Table 2).

**Table 2:** Risk Factors for Venous Thromboembolism

Risk Factors	N (%)
Obesity	53 (53)
Recent surgery	15 (15)
Family history of VTE	9 (9)
Thrombophilia	8 (8)
Immobilization	8 (8)
Previous VTE	7 (7)
Diabetes	5 (5)
Recent hospitalization	4 (4)
Hypertension	2 (2)
Crohn’s disease	1 (1)
Nephrotic syndrome	1 (1)
Cancer	1 (1)
Other	12 (12)
Trauma	3 (3)
Abortion	3 (3)
Pulmonary hypertension	2 (2)
Seizure disorder	1 (1)
Post-partum	1 (1)
Splint fracture	1 (1)

**Figure 1:**
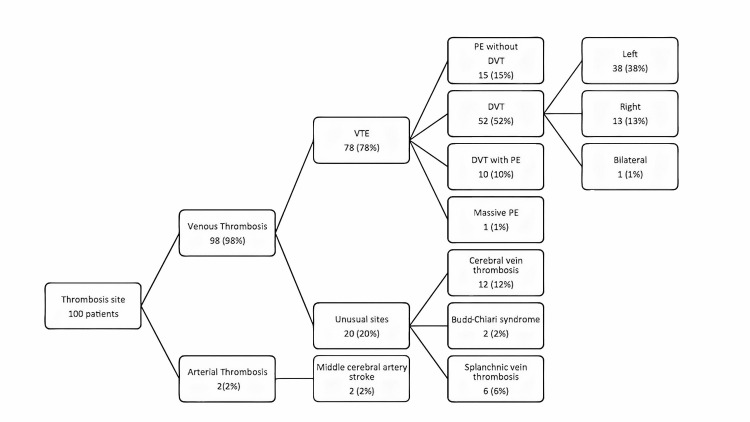
Thrombosis Site

Of the patients, 78% experienced a VTE (Figure 1), 10% with VTE also had a PE, 10% with a DVT also had a PE, and 52% were diagnosed with DVT alone; 38 (38%) of these occurred in the left leg, 13 (13%) in the right leg, and one (1%) in both legs. Unusual sites of VT included cerebral vein thrombosis (n=12, 12%), Budd-Chiari syndrome (n=2, 2%), and splanchnic thrombosis (n=6, 6%). Two (2%) patients experienced a middle cerebral artery stroke, without any vascular risk factor other than COC use. Patients were treated with warfarin (n=64, 64%), rivaroxaban (n=32, 32%), and other antithrombotic medications (n=4, 4%). Of the patients, 94% completed their VTE/PE therapy.

## Discussion

The reported risks for vascular thrombosis in the general population include major surgery, major trauma, hip fracture, and lower extremity paralysis secondary to spinal cord injury [18]. Previous thrombotic events, advanced age, cardiac or respiratory failure, prolonged immobility, estrogen use, and thrombophilia have been associated with a lesser increased risk for VTE events. This is the first descriptive study conducted in Saudi Arabia characterizing patients with COC use and vascular thrombosis.

Venous thrombosis has been reported to occur in the general population at a rate of 0.16 to 0.37 per 1,000 person-years [19]. The increased risk for VTE associated with COC use has been reported as 3.5, compared to patients not taking contraceptives. The increased risk of venous thrombosis was reported with first-generation (3.2-fold), second-generation (2.8-fold), and third-generation (3.8-fold) progestogens. 50LNG users had the highest risk of venous thrombosis; 30DRSP, 35CPA, and 30DSG users had a similar middle risk; and 30LNG, 20LNG, and 20GSD users had the lowest risk. According to the MEGA case-control study conducted in 2009, the VTE risk linked to the progestin component in COCs was the highest in the third- and fourth-generation contraceptive pills [6].

Patients older than 40 years of age are at increased risk of thrombotic or embolic events [18]. Women we saw taking COCs had a similar age range. The association of obesity with vascular events is not well defined [18,20]. Of the women we examined, 53% were obese or morbidly obese. A cohort study found that obese women who used COCs had a 24-fold higher thrombotic risk than women with a normal BMI who did not use COCs [21].

A retrospective review of medical records from 2,218 patients from Olmsted County, Minnesota, USA, identified an overall average age- and sex-adjusted annual incidence of VTE/PE of 117 per 100,000 (DVT, 48 per 100,000; PE, 69 per 100,000). The incidence of VTE/PE in the general population increased with age. Patients treated in the hospital had three-year mortality of 30% [18,22]. A multicenter study evaluated 1,103 Dutch women who developed VTE while taking COCs. They found that the risk of deep venous thrombosis in women taking COCs increased with increasing age (<30 years, RR=3.7; 30-40 years, RR=10; 40-50 years, RR=13.3) [6]. The relative risk was higher for women with a DVT of the leg (odds ratio=6.6) than for women with a PE, with or without DVT of the leg (odds ratio=3.9). The odds ratio was not affected by the type of progestogen found in the COC.

The odds ratio of DVT has been reported to be highest during the first three months of taking COCs, which is similar to our patients [6]. The reported patients have a similar distribution of duration of treatment with COCs as the patients we treated. Inherited thrombophilic syndromes are well-described risk factors for VTE [18]. The women we treated with VTE had a similar incidence of thrombophilia as patients not taking COCs.

A broader problem with unusual sites of thrombotic disease was identified in this study, compared to previous studies. The unusual sites of VTE included cerebral vein thrombosis (n=12, 12%), Budd-Chiari syndrome (n=2, 2%), and splanchnic thrombosis (n=6, 6%) [23-27]. We also found two arterial ischemic strokes, which is consistent with previously published studies [9,10].

There were several limitations to this study. This retrospective study was dependent on data available from chart review and on the legibility of handwriting in the chart. Since thrombophilia screening is expensive and time-consuming, not all patients underwent a thrombophilia workup. There was no control group to compare outcomes and assess risk.

## Conclusions

This study characterizes Saudi women with thrombotic events taking COCs and identifies risk factors, including unusual sites of thrombosis. Most patients experienced the vascular event during the first year of taking COCs. Age of 40-50 years, obesity, and thrombophilia were the commonly observed risk factors. Patients taking COCs need counseling on the risk of thrombosis while taking COCs. A case-control study is needed to determine the risk of vascular thrombosis with COC use in Saudi Arabia.
